# Teaching About “Brain and Learning” in High School Biology Classes: Effects on Teachers' Knowledge and Students' Theory of Intelligence

**DOI:** 10.3389/fpsyg.2015.01848

**Published:** 2015-12-01

**Authors:** Sanne Dekker, Jelle Jolles

**Affiliations:** ^1^Department of Educational Neuroscience, Faculty of Behavioural and Movement Sciences, VU University AmsterdamAmsterdam, Netherlands; ^2^Science Hub Radboud University, Institute for Science, Innovation and Society, Faculty of Science, Radboud UniversityNijmegen, Netherlands

**Keywords:** brain and learning, educational neuropsychology, teaching module, biology, theory of intelligence

## Abstract

This study evaluated a new teaching module about “Brain and Learning” using a controlled design. The module was implemented in high school biology classes and comprised three lessons: (1) brain processes underlying learning; (2) neuropsychological development during adolescence; and (3) lifestyle factors that influence learning performance. Participants were 32 biology teachers who were interested in “Brain and Learning” and 1241 students in grades 8–9. Teachers' knowledge and students' beliefs about learning potential were examined using online questionnaires. Results indicated that before intervention, biology teachers were significantly less familiar with how the brain functions and develops than with its structure and with basic neuroscientific concepts (46 vs. 75% correct answers). After intervention, teachers' knowledge of “Brain and Learning” had significantly increased (64%), and more students believed that intelligence is malleable (incremental theory). This emphasizes the potential value of a short teaching module, both for improving biology teachers' insights into “Brain and Learning,” and for changing students' beliefs about intelligence.

## Introduction

Knowledge of the brain's development and its involvement in the learning process has rapidly increased in the last decade. These new insights may have relevance for educational practice (e.g., OECD, 2002, 2007; Jolles et al., 2005; Howard-Jones, 2010; Spitzer, 2012). For example, learning about brain and neuropsychological development in adolescents may increase teachers' understanding of typical adolescent behavior such as risk taking, and offer them insight into the ongoing development of neuropsychological functions such as self-evaluation, monitoring, and planning. This may positively influence teachers' patience and optimism, as well as helping them to develop an effective professional attitude toward students (Hook and Farah, 2012). Currently, there is a need for scientifically validated courses about the brain and its functions for school settings (OECD, 2002, 2007). In the present study, we evaluated the effects on teachers and students of a newly developed teaching module about “Brain and Learning” using a controlled design.

In general, there is a growing interest among teachers to learn more about the brain (Pickering and Howard-Jones, 2007; Serpati and Loughan, 2012). Particularly science teachers are eager to embed topics related to neuroscience in their curricula (Dubinsky, 2010). However, current biology textbooks only cover the basic aspects of neuroscience. They are generally limited to topics from classical biology, like the biology of the senses and the human nervous system, or the neurobiological and physiological processes underlying neuronal transmission (e.g., Waas, 2009). Information related to the functions of the brain is seldom a topic in high school biology classes. For instance, one of the subjects rarely addressed is that higher-order cognitive processes continue to develop into late adolescence, and that the environment plays a major role in providing the context for brain development and maturation. This raises the question how well biology teachers are acquainted with the more “functional” aspects of the brain and with its involvement in learning.

In the field of educational neuropsychology, it has been argued that knowledge of the more “functional” aspects of the brain is relevant for educational practice (Jolles et al., 2006; Fischer et al., 2007; Spitzer, 2012). If teachers know that the underlying brain networks for planning abilities continue to mature during adolescence, and that this development is contingent upon experiences, they will understand that they have to provide more guidance in order to stimulate the development of students' planning abilities. Likewise, understanding the processes underlying learning and memory formation may help teachers to improve classroom practices (Dommett et al., 2011). For example, when teachers understand that learning involves the formation of strong connections within networks of neurons in the brain, and that brain connections are strengthened by rehearsal, they may make more effort to rehearse frequently in their lessons. Thus, this knowledge may positively influence the teachers' pedagogical approach.

Similarly, students may benefit from this knowledge, when they realize that learning is an active process where performance is amenable to improvement, and that effort, training, and rehearsal are crucial for the outcome of the learning process. According to the so-called “theory of intelligence” (TOI), understanding the concept of brain plasticity can alter students' implicit beliefs about learning potential (Dweck, 1986; Blackwell et al., 2007). Intelligence may either be viewed as a fixed quantity which one has little influence over (referred to as “entity theory”), or as a malleable trait that can be improved by effort (referred to as “incremental theory”). Understanding that the brain is shaped by experience promotes an incremental theory (Dweck, 1986; Blackwell et al., 2007). Evidence is accumulating that an incremental theory can be taught and that this has a positive effect on persistence (O'Rourke et al., 2014; Renaud-Dubé et al., 2015). Furthermore, holding an incremental theory has been related to higher school motivation and better student achievement (Blackwell et al., 2007). Interventions as short as 3 min were proven to be sufficient to reveal positive effects (O'Rourke et al., 2014). Thus, next to enhancing teachers' knowledge and competence, new insights about the brain and its involvement in learning may also be beneficial for students.

Therefore, this study focuses on the evaluation of a newly developed teaching module about “Brain and Learning.” This teaching module comprised three lessons: (1) brain processes underlying learning; (2) neuropsychological development during adolescence; and (3) lifestyle factors that can influence learning performance. The module was implemented in high school biology classes for grades 8–9 by teachers who were interested in the brain's involvement in learning. The first aim was to examine how well biology teachers were acquainted with the more “functional” aspects of the brain and with the brain's involvement in learning (in short: “Brain and Learning”). Secondly, the study aims to enhance teachers' knowledge of “Brain and Learning” and to teach students an incremental TOI using a controlled and matched group design. The hypotheses were: (1) before intervention, teachers' knowledge of “Brain and Learning” is significantly lower than knowledge of basic neuroscience concepts; (2) teachers who implemented the teaching module have more knowledge of “Brain and Learning” than teachers in the waiting-list control group; and (3) incremental theories of intelligence are more prevalent among students in the intervention group than among students in the waiting-list control group.

## Materials and methods

### Participants

A total number of 41 biology teachers and 1241 students in grades 8–9 from schools across the Netherlands participated in this research project. The intervention group comprised 18 teachers and 456 students. The waiting-list control group comprised 23 teachers and 785 students. To minimize any possible confounding effects of teachers' background characteristics, teachers in both groups were matched on age, sex, education level, and teaching experience. This resulted in a final sample of 32 teachers, i.e., 16 teachers in each group. All teachers indicated that they were interested in the brain and how it is involved in learning. There were no significant between-group differences in teachers' prior knowledge of “Brain and Learning” (see Table 1). In the student sample, the *M* age was 14.5 (*SD* = 0.65) and the sample consisted of 46% boys. There were no significant between-group differences with respect to students' age [*M* = 14.5; *SD* = 0.65; *t*_(1200)_ = 1.14, *p* = 0.256] and sex [χ^2^(1, *N* = 1239) = 0.385, *p* = 0.535]. The sample was homogeneous with respect to educational track. All teachers and students were in high educational tracks, which prepare students for higher tertiary education like professional education programs and/or university. The incidence of problem behavior (e.g., externalizing problems or problems with peers) in these tracks is much lower than in educational tracks that prepare for vocational training programs (van Dorsselaer et al., 2010). Therefore, possible confounding effects of problematic student behavior were minimized.

**Table 1:** **Teacher characteristics**.

	**Intervention group**	**Control group**	**Test statistic**
*N*	16	16	
Age M(SD)	43.7 (10.0)	45.6 (11.3)	*t*_(30)_ = 0.514, *p* = 0.611
Years teaching *M*(SD)	14.6 (11.0)	15.1 (9.8)	*t*_(30)_ = 0.152, *p* = 0.880
Sex			χ^2^(1, *N* = 32) = 1.00, *p* = 1.00
Male	38%	38%	
Female	62%	62%	
Education level			χ^2^(1, *N* = 32) = 0.130, *p* = 0.719
Higher education	44%	38%	
University	56%	63%	
Attended at least 1 lecture about “Brain and Learning” before participation	62%	38%	χ^2^(1, *N* = 32) = 2.00, *p* = 0.144
Read at least 1 popular book about “Brain and Learning” before participation	44%	38%	χ^2^(1, *N* = 32) = 0.130, *p* = 0.500

### Procedure

The schools that were approached to participate in this study can be considered a random selection of secondary schools distributed across the Netherlands. Emails were sent to the schools administration, with the request to forward information about the research project to their biology teachers. Biology teachers who were interested in participating followed a link to an online registration form where they provided contact information and indicated their availability for two research periods in the forthcoming 2 months. After at least 40 teachers had signed up for participation, the registration form was closed. The teachers selected one or more of their classes in which to implement the module. Thus, the student selection was determined by the teachers. Students were informed about the project by information letters sent to their home address. All students in the selected classes received the lessons. If informed consent was given by the students and their parents, students also participated in the research project and data collection.

**Figure 1:**
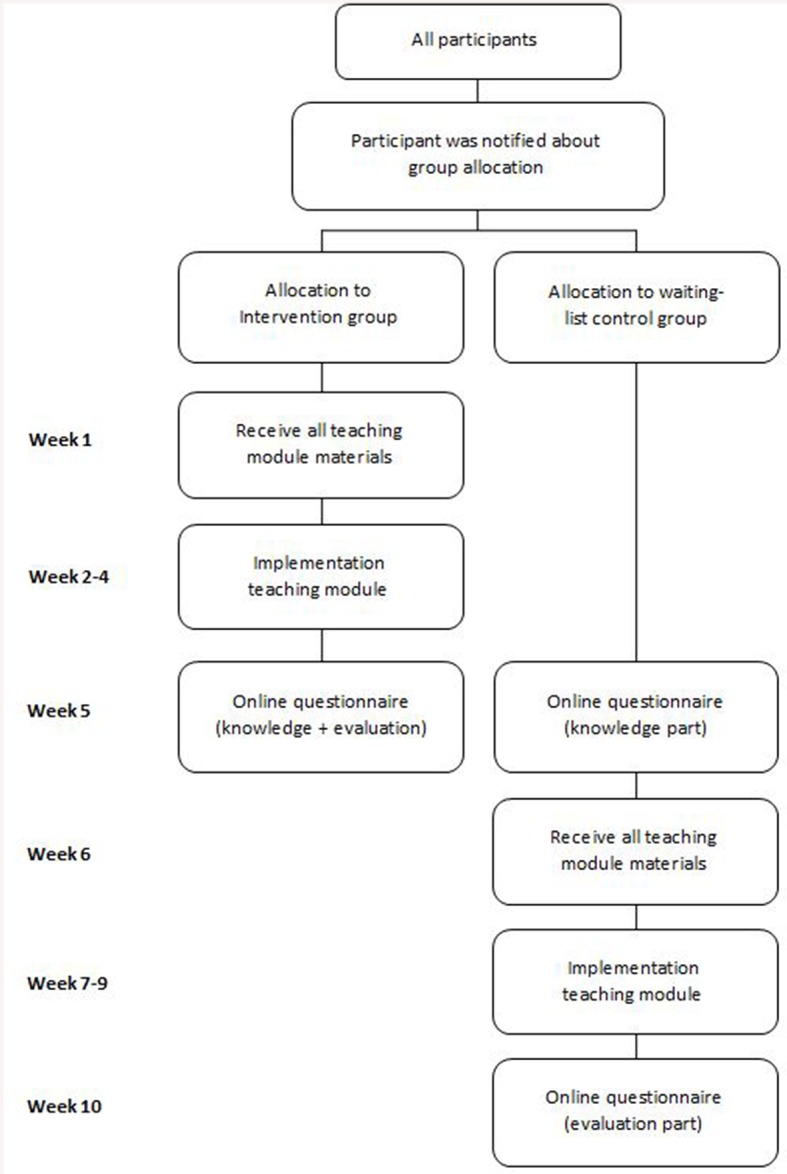
**Study design**.

The design of the study is schematically represented in Figure 1. Teachers were allocated to either the intervention or the waiting-list control group. The time of their availability played a role in allocating teachers to a group. Teachers who signed up for the first research period were assigned to the intervention group. The remaining teachers were assigned to the waiting-list control group. All participants were blind for group allocation. Teachers at the same school always participated in the same research period. If teachers at the same school were not available for the same period, they were contacted and asked to decide upon the research period that fitted them both. This preempted consultation between teachers in the intervention and waiting-list control group. After group allocation teachers either started the project directly (intervention group), or waited several weeks before starting (waiting-list control group).

At the start of the project, teachers received an information letter and the required materials. The letter informed them of planning and procedure, as well as describing what was expected of them. Lesson materials included an extensive protocol, supplementary Powerpoint Presentations, and in-depth background information. Furthermore, teachers received access to an online discussion forum. This forum facilitated communication with fellow participants and with the researchers who had developed the teaching module. All teaching materials and suggestions for further reading were available on this forum.

Assessment took place after the intervention group had been taught the teaching module, and before the waiting-list control group received any of the materials. For both teachers and students, assessment consisted of completion of an online questionnaire. For teachers, this took about 20 min to complete; students needed about 10 min. Teachers and students from the control group completed the evaluation part of the assessment after implementation of the lessons.

The study was performed under the ethical guidelines of VU University Amsterdam. With respect to approval by an Ethical Committee, the project falls under the umbrella of a large-scale research program into the effects of educational interventions based on neuropsychological insights, for which approval has been obtained (BREIN, April 2010). Both adolescents and parents were required to give written informed consent in order to participate in this research. The intervention only comprised relevant knowledge and insights about biology and neuropsychology. Nor educational materials, nor any of the evaluation measures related to the participants' psychological wellbeing, medical history or socio-economic background. Because the research contained no elements with potential influence on psychological wellbeing or other psychological functions, additional ethical approval was not required for this specific project.

### Measures

#### Background characteristics

Both teachers and students completed a questionnaire about background characteristics, including demographic variables. Teachers additionally reported interest in “Brain and Learning” and whether they had attended lectures or read books on this topic.

#### Evaluation of the teaching module

Both teachers and students evaluated the teaching module and reported their experiences with the program. Relevant topics were: interest and appraisal of the teaching module, quality of the teaching module, reactions to the teaching module, and general feedback on the teaching module. Additionally, teachers were asked about adherence to the protocol.

#### Knowledge questionnaire (for teachers)

A questionnaire was developed to assess teachers' knowledge. The questionnaire comprised 16 questions about the various brain functions and brain development (based on information presented in the teaching module) and 10 questions about basic neuroscientific concepts (based on themes included in the standard biology curriculum). The order of the questions was randomized. There were forced-choice questions (true, not true, I don't know), multiple choice questions, and open-ended questions. Teachers received 1 point for each question that was answered correctly. Sum scores were calculated for knowledge of brain functions [range 0–16], and knowledge of basic neuroscience [range 0–10], separately. Dependent variables were: the percentage of correct answers on questions about brain functions; and the percentage of correct answers on basic neuroscience questions.

#### Theory of intelligence (for students)

To assess the students' TOI, students were asked to choose from four statements regarding the ability to change intelligence. They had to complete the following sentence about intelligence: “You are born with a certain amount of intelligence….” Answer options were: (1) and you can't do anything to change it (strong entity theory); (2) and you can do little to change it (moderate entity theory); (3) but you can change to some extent how intelligent you are (moderate incremental theory); or (4) but you can always change how intelligent you are (strong incremental theory). These statements were based on previous work by Dweck (1986).

### Teaching module

The teaching module about “Brain and Learning” comprised the following three 45-min lessons: (1) general information about the brain in relation to learning and memory; (2) the development of the brain during adolescence and its consequences for adolescent behavior (e.g., risk-taking, impulsiveness, sensitivity to peer-pressure) and learning; and (3) the consequences of biopsychological factors and behavioral habits on the brain (e.g., alcohol, nutrition, sleep, stress, physical exercise). A summary of the content of the lessons can be found in the Supplementary Material. A protocol was used to structure each lesson, which included information, assignments, movie fragments, and interesting facts. To control for differences between teachers, “main points” were defined for each lesson and it was obligatory for teachers to discuss these with their students. Next to the lesson protocol, there was an additional manual for teachers containing more in-depth information, the explication of neuromyths, and suggestions how they could use the findings to improve their teaching practice. For example, they were advised to bring variation into their lessons to keep the students' attention.

### Data analyses

All data processing took place using the Statistical Package for the Social Sciences (SPSS) v.20 for Windows. Before analyzing the data, teachers in both groups were matched on age, sex, education level, and teaching experience to decrease the between-group variance as much as possible. In this way, the strengths of a within-group design (no confounding of differences between participants) and between-group design (no learning effects on the knowledge questionnaire) were combined. All data analyses related to the teachers were performed on the matched sample. First, teachers' knowledge in the control group was assessed (before intervention). A paired *t*-test was used to assess differences between knowledge of brain functions and development, and knowledge of basic neuroscience. Next, teachers in the intervention group were compared to the control group to examine the effects of the intervention on teachers' knowledge. An independent *t*-test was used to examine differences in knowledge of brain functions and development (dependent variable) between the intervention and the waiting-list control group. Third, a regression analysis was performed on the teacher sample to examine which factors predicted their knowledge of brain functions and development. Predictors were: intervention group (0 = control, 1 = intervention); sex (0 = male, 1 = female); education level (0 = higher professional education (applied sciences), 1 = university); years of teaching; prior knowledge of brain and learning topics: lecture attendance (0 vs. 1 or more lectures); prior knowledge: books about the topic (0 vs. 1 or more books); and percentage correct answers on basic neuroscience questions.

For student outcomes, the four answer options for TOI were: strong entity (1); moderate entity (2); moderate incremental (3); and strong incremental (4). A chi-square test was used to examine whether the distribution of theory of intelligence differed between groups.

After these analyses, we assessed how the teaching module was evaluated by all teachers and students who had participated in this research. Evaluation data of 35 teachers and 893 students from the original sample were available. The statistical threshold was α = 0.05.

## Results

### Teachers' knowledge

#### Knowledge before intervention

Prior knowledge was assessed in teachers who did not yet have access to any of the teaching materials (waiting-list control group, *N* = 16). The teachers answered 46% of the questions about brain functions and brain development correctly. This was significantly lower [*t*_(15)_ = −5.64, *p* < 0.000] than performance on basic neuroscientific knowledge, where they answered 75% of the questions correctly. This indicates that teachers' knowledge of how the brain functions and develops was significantly lower than their knowledge of basic neuroscientific topics.

#### Knowledge after intervention

An independent *t*-test showed that knowledge of brain functions and brain development was significantly higher in the intervention group than in the control group, [*t*_(30)_ = 4.29, *p* < 0.000]. Teachers who had taught the module answered 64% of the questions correctly on average, compared to 46% in the control group. Basic neuroscientific knowledge did not differ significantly between groups, [*t*_(30)_ = 1.56, *p* = 0.130; see Table 2].

**Table 2:** **Percentage of correct answers**.

	**Intervention group *M (SD)***	**Control group *M (SD)***	**Test statistic**
Knowledge of brain functions and brain development	64% (12.1)	46% (12.0)	*t*_(30)_ = 4.29, *p* < 0.000
Basic neuroscientific knowledge	84% (13.1)	75% (18.3)	*t*_(30)_ = 1.56, *p* = 0.130

#### Predictors of knowledge of brain functions and brain development

A regression analysis showed that knowledge of how the brain functions and develops was predicted by intervention group (β = 0.56) and by prior knowledge as a consequence of lectures (β = 0.39; see Table 3). This indicates that knowledge of brain functions and brain development was higher in the intervention group than in the control group. Furthermore, knowledge was higher among participants who had attended one or more formal presentations (e.g., seminars, workshops) about “Brain and Learning” outside this study. Knowledge was not predicted by sex, education level, number of years teaching, reading popular books about the topic, or basic neuroscientific knowledge. The model explained a significant proportion of variance (*R*^2^ = 0.59) in knowledge, *F*_(7, 24)_ = 4.86, *p* = 0.002.

**Table 3:** **Predictors of knowledge of brain functions and brain development**.

	***B* (*SE*)**	***t***	***p***	**95% CI for *B***	
				**Lower**	**Upper**
Intercept	0.433 (0.114)	3.80	0.001	0.198	0.669
Intervention group	0.165 (0.043)	3.82	0.001^**^	0.076	0.255
Sex	0.056 (0.046)	1.22	0.235	−0.039	0.151
Educational level	0.074 (0.053)	1.39	0.178	−0.036	0.184
Years teaching	−0.003 (0.002)	−1.25	0.223	−0.008	0.002
Prior knowledge: lectures	0.115 (0.045)	2.56	0.017^*^	0.022	0.208
Prior knowledge: books	−0.042 (0.045)	−0.945	0.354	−0.135	0.050
Basic neuroscience knowledge	−0.056 (0.168)	−0.332	0.743	−0.402	0.291

* p < 0.05,

** p < 0.01.

### Student outcome: theory of intelligence

**Figure 2:**
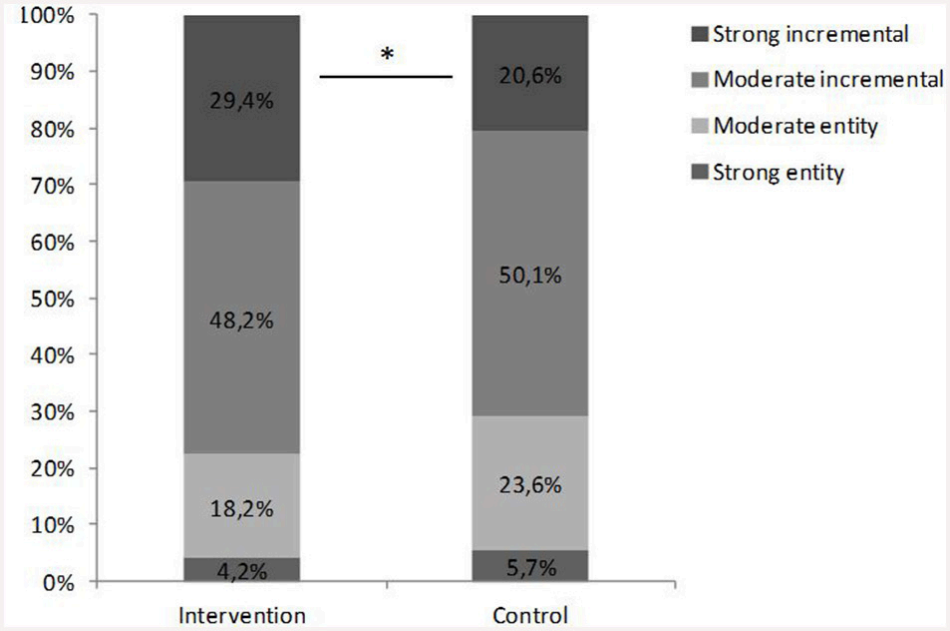
**Distribution of TOI among the control and intervention group**. ^*^p < 0.05.

The distribution over four types of TOI (strong entity, moderate entity, moderate incremental, strong incremental) was examined for both groups using Chi-square tests. A significant between-group difference was found in TOI, $\chi_{\left(3\right)}^{2}=14.7$, *p* = 0.002. Figure 2 displays the distribution of the different theories for both the intervention and the control group. The standardized residuals of each category showed that only the strong incremental view was more frequent in the intervention group (29%, *z* = 2.4, *p* < 0.05) than in the control group (21%, *z* = −1.8, *p* > 0.05). Thus, students were more likely to hold a strong incremental theory when they had been taught a module that addressed brain plasticity. The numbers indicate that without intervention, 4 out of 20 students think that intelligence is very malleable and changeable through experience. After intervention, this increases to 6 out of 20 students. There were no significant group differences for the moderate incremental or entity categories.

### Subjective evaluation of the teaching module

#### Teacher evaluation (N = 35)

Almost all teachers (*N* = 33; 94%) found the lessons informative for students. Sixty percent of the teachers (*N* = 21) rated the quality of the information in the teaching module as “high”; another 37% as “average” (*N* = 13); and 3% as “poor” (*N* = 1). Furthermore, a large majority (*N* = 31; 89%) indicated that students reacted positively to the lessons. The attractiveness of the module (whether it was fun and interesting) was rated as: “high” by 46% of the teachers (*N* = 16); “average” by 43% (*N* = 15); and “poor” by 11% of the sample (*N* = 4). Teachers were not sure whether the lessons stimulated their students' school motivation. Improvements in school motivation were mentioned by 11% of the teachers (*N* = 4). The data of teachers' final rating of the teaching module [range: 1–10] were negatively skewed, with a median of 7.0. Teachers mainly recommended increasing the number of assignments for students, and decreasing the amount of information. Most of the teachers (80%) would recommend the module to other teachers. Additionally, 89% of them would encourage the incorporation of this module into the standard biology curriculum.

#### Student evaluation (N = 893)

Most students (81%) would recommend the teaching module to other schools. They were most satisfied about the amount and clarity of the information. One third of the students were eager for more tips for learning. The module could be improved to be more useful for learning according to 56% of the students. The majority of the students (65%) recommended improving the attractiveness of the materials, for instance by including more movies and/or other media.

## Discussion

This study examined the effect of a “Brain and Learning” teaching module on biology teachers' knowledge and on their high school students' theory of intelligence (TOI). Before intervention, the biology teachers' knowledge of brain functions and brain development was significantly lower than knowledge of basic neuroscientific concepts (46 vs. 75% correct answers). General knowledge of basic neuroscientific concepts did not predict knowledge of how the brain functions and develops. Yet, teachers who had attended at least one formal presentation (seminar, workshop) about “Brain and Learning” before participation in this study knew more than teachers who had not attended any lectures. Furthermore, teachers who had taught the newly developed module knew more than teachers in the control group. With respect to students' TOI, we found that only the strong incremental theories were more frequent in the intervention group than in the control group. The findings suggest that the teaching module was effective in enhancing biology teachers' knowledge of brain functions and brain development, and indicate that it could promote a strong incremental TOI in students.

Our results confirm the hypothesis that teachers who are interested in the brain and its involvement in learning are not very familiar with the topic. This was previously found to be the case with trainee teachers in the UK (Howard-Jones et al., 2009) and with teachers interested in the neuroscience of learning (Dekker et al., 2012). A lack of familiarity with the topic may be due to a lack of training, as new insights about how the brain functions and develops obtained in the past decade are currently not embedded in teacher training. This research project showed that knowledge was improved when biology teachers had attended one or more lectures about “Brain and Learning.” This is congruent with previous research showing that neuroscience workshops led to increases in teachers' neuroscientific knowledge (MacNabb et al., 2006; Dommett et al., 2011). The findings stress the importance of developing student and teacher trainings based on neuropsychological or biopsychological insights. Furthermore, our study showed that, in the case of biology teachers, knowledge and insights were positively predicted by teaching a module on this topic. This suggests that the teaching module can be a successful continuing education program with which to stimulate biology teachers to improve their knowledge of the latest results in the field of behavioral neuroscience.

Furthermore, the intervention changed some students' beliefs about intelligence: students who had learned about brain plasticity and knew that the brain is shaped by experiences more often held a strong incremental TOI. Yet, the practical relevance of this finding should be interpreted with caution, because the percentage of students who held strong incremental beliefs after intervention was still quite small (29%). Previously, Blackwell et al. (2007) showed that an intervention about brain plasticity can influence students to adopt an incremental theory of intelligence. The content of their intervention was more focused on TOI and comprised eight lessons of 25 min, instead of three lessons of 45 min. This suggests that interventions may be more effective in changing students' beliefs about intelligence when they are more intense than the module described in this study. As positive attitudes toward school underlie successful school performance, future research should focus on promoting these adaptive student attitudes.

The study showed that it was feasible to embed a teaching module in the current biology curriculum. According to the teachers and students involved, this teaching module could make a valuable contribution to the current high school biology curriculum. The present study has been set up as a “feasibility study”. The majority of teachers and students thought that the lessons were informative and of good quality. Nevertheless, the teaching module can still be improved. Both teachers and students made suggestions for improving the attractiveness of the materials of the teaching module. Ideally, biology teachers themselves should be involved in this process (Dommett et al., 2011). Furthermore, future research could focus on more long-term outcomes of implementation of the teaching module (e.g., student achievement).

The current results about teachers' knowledge only reflect the performance of teachers who are interested in how the brain is involved in learning. Poor familiarity with the topic may be of particular concern in this group, because findings show that these teachers will be most likely to implement their (wrong) ideas in practice (Dekker et al., 2012). Another point of consideration is that scores may be somewhat different in the general population of high school biology teachers. Probably, knowledge of “Brain and Learning” will be even lower in the case of biology teachers who are less interested in this topic. On a final note, we must be careful to draw conclusions about causality, as we did not measure within-group changes in knowledge and TOI over time. Future research is needed to address this issue.

In conclusion, this study showed that our teaching module about “Brain and Learning” was a successful continuing education program with which to stimulate biology teachers to improve their knowledge of the latest results in the field of behavioral neuroscience. Furthermore, there are indications that students are more likely to hold a strong incremental theory after being taught this module, which in turn has previously been related to improvements in academic achievement. Therefore, we argue that teaching modules like the one described in this article can be a valuable addition for the current high school biology curriculum. It can contribute to a successful integration of neuroscientific insights in education and may consequently improve the quality of education.

### Conflict of interest statement

The authors declare that the research was conducted in the absence of any commercial or financial relationships that could be construed as a potential conflict of interest.
