# Supplementary material for: Teaching About “Brain and Learning” in High School Biology Classes: Effects on Teachers' Knowledge and Students' Theory of Intelligence
Source: Front Psychol. 2015 Dec 1;6:1848. doi: 10.3389/fpsyg.2015.01848 (PMC4664647; doi:10.3389/fpsyg.2015.01848)
Supplement: Supplementary file 1 [file DataSheet1.DOCX]

**Appendix: *Summary of the teaching module***

**Lesson 1: The brain**

Students obtained a general knowledge of basic brain functions and learning principles.

*Core concepts*

- The brain consists of many highly-connected cells, that communicate with each other
- Learning involves the creation of new connections between nerve cells in your brain
- Memory processes are responsible for information storage
- Repetition enhances the strengthening of connections in your brain
- Attention is necessary for learning
- Your brain categorizes incoming information
- The structure of your brain changes as a result of learning

The in-depth information for teachers included information about the nervous system, brain anatomy and general brain functions. Also, more information was given about memory and the biological processes involved (e.g., LTP). Practical tips were included about rehearsal, the role of attention in learning, and connecting new information to existing knowledge.

**Lesson 2: The adolescent brain**

Students gained understanding of changes in brain and behavior during adolescence

*Core concepts:*

- Many brain and neuropsychological changes occur during adolescence
- The emotion and control systems are out of balance during adolescence
- Adolescents often *act* first, and *think* later
- Adolescents often take more risks than adults
- Rewards are important for adolescents
- Due to brain maturation processes, it can be difficult to control your emotions during adolescence
- The development of the brain differs between individuals

The in-depth information for teachers included information about adolescent brain development. It focused on the development of the limbic system and the prefrontal cortex. It explained how the different pace of development in these regions can give rise to typical adolescent behaviors (impulsiveness, risk-taking behavior, reward sensitivity, peer-pressure). Practical tips were included about feedback and judgments of behavior.

**Lesson 3: Good for your brain**

Students were made aware that lifestyle has an influence on the brain.

*Core concepts:*

- Environmental factors play an important role in brain development (i.e. the influence of parents, teachers, context and also biopsychological factors related to incoming information (senses) and information regarding processes related to sleep, nutrition, etc)
- Breakfast is important for learning
- The sleeping pattern changes during puberty. It is best to keep a daily routine.
- Motor exercise and experience in ‘action’ has a positive influence on the brain
- The influence of stress on learning
- Drugs and alcohol influence brain functioning
- Energy drinks contain too much caffeine

The in-depth information for teachers included an elaboration of the mechanisms underlying environmental influences on the brain. Practical tips were about increasing students’ awareness of their lifestyle.
